# Structural Colors from Modulated Topological Defects in Molecular Smectic Liquid Crystals

**DOI:** 10.1002/advs.76646

**Published:** 2026-07-30

**Authors:** Camille N. Mahyaoui, Guilhem Poy, Rabab Zehra, Axel Fouques, Vassili Sergan, Ivan Dozov, Claire Meyer, Patrick Davidson

**Affiliations:** ^1^ Saint‐Gobain Recherche Paris Aubervilliers France; ^2^ Laboratoire De Physique Des Solides Université Paris Saclay CNRS Orsay France; ^3^ L2C, Univ. Montpellier CNRS Montpellier France; ^4^ Physics and Astronomy California State University Sacramento California USA; ^5^ Physique Des Systèmes Complexes Université De Picardie Jules Verne Amiens France

**Keywords:** liquid crystals, smectic, structural colors, topological defects

## Abstract

Unlike absorption colors, which are produced by light‐absorbing dyes and pigments, structural colors arise from the wavelength‐dependent interference of light scattered by microstructures produced through the self‐organization of soft matter systems, like colloids or chiral liquid crystals. However, applications of structural colors require forming structures with micron‐scale periodicities that are well‐ordered and defect‐free over large areas, which can only be achieved through complex sample processing methods. Here, we demonstrate another approach based on the striated linear topological defects appearing in thin liquid crystal smectic films under hybrid anchoring conditions. We produced structural colors in both reflection and transmission geometries simply by spin‐coating the widespread commercial achiral molecular liquid crystal 8CB over glass slides. Optical microscopy, AFM, and optical diffraction techniques show that the colors are due to the modulation period along the defect axis being comparable to the wavelengths of visible light. Our approach dispenses with the tedious synthesis or purification of chiral organic compounds and with achieving ideal periodic microstructures because it relies on the spontaneous formation and uniform modulation of topological defects, allowing for upscaling to the large areas required for applications.

## Graphical abstract

Rather than using dyes and pigments, nature produces the colors of some butterflies and beetles through optical interference phenomena. This study describes an original biomimetic strategy exploiting the spontaneous formation of modulated linear defects in thin films of 8CB, a common molecular liquid crystal. This avoids the synthesis and purification procedures of current attempts to reproduce this phenomenon using liquid crystal organizations.

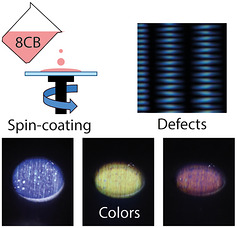

## 1 Introduction

The sensation of color is produced in two different ways, in industry as in nature. The first way relies on the use of special chemicals called dyes and pigments that absorb part of the spectrum of visible light. Dyes are organic molecules that require costly synthesis or purification from natural products and that are often sensitive to light‐induced degradation, both in the UV and visible wavelength range, all the more since they are selected for their strong light‐absorption properties. Pigments are minerals that require high‐temperature chemical synthesis or must be mined from the earth using polluting industrial processes. The second way relies on the wavelength‐dependent light interference phenomena produced by microstructured materials, resulting in so‐called “structural colors” [1]. The microstructures can be created by “top–down” techniques, such as microlithography, which sculpt matter at the micrometer scale, or by “bottom–up” methods based on the self‐assembly of colloidal particles [2, 3, 4] or the spontaneous large‐scale periodic organization of molecules, such as liquid crystalline compounds [5, 6, 7]. In the latter case, structural colors are most often produced by exploiting the helical structure, with a pitch close to the wavelength range of visible light, of the cholesteric phase formed by chiral molecules or polymers [8, 9, 10, 11, 12, 13, 14, 15]. However, in addition to quite complicated processes of sample preparation, the chiral liquid crystal materials themselves can only be obtained by costly organic asymmetric synthesis or by time‐consuming purification and acid hydrolysis of natural compounds (mostly cellulose and chitin). Structural colors have also sometimes been observed in smectic phases of colloidal suspensions of anisotropic mineral nanoparticles, due to their large lamellar periods [16, 17, 18, 19, 20, 21]. However, these mineral particles are generally prepared by high‐temperature solid‐state chemistry techniques, and achieving their colloidal stability in suspension is a delicate step.

We describe here an alternative approach to the production of structural colors using liquid crystals. Instead of relying on the self‐assembly of perfectly ordered (i.e., defect‐free) structures with large periods, our approach exploits the spontaneous formation of topological defects in thin smectic A liquid crystal films of achiral molecules subjected to “hybrid” anchoring conditions (described below). Because the smectic A phase of molecular liquid crystals has a period of typically a few nanometers, its layered structure cannot give rise to structural colors, which must then arise from textural properties. The textures of smectic films have already been studied in detail both at very low film thicknesses, where uniform linear topological defects are observed, and at larger thicknesses, where focal conic domains predominate, but the intermediate thickness (≈ 1 µm) regime has attracted less attention [22, 23, 24, 25, 26, 27]. Here we show that these smectic films produce structural colors because their linear defects are modulated along their axis with a period of about a micron, which can be easily tuned by adjusting the film thickness. These structural colors are iridescent, and their intensity also strongly depends on the viewing direction with respect to the planar anchoring direction of the liquid crystal. Most importantly, they are obtained simply by spin‐coating a small amount of the most common commercial smectic liquid crystal, called “8CB,” onto glass slides.

## 2 Results

### 2.1 Qualitative Approach to the Optical Properties of the Samples

**FIGURE 1:**
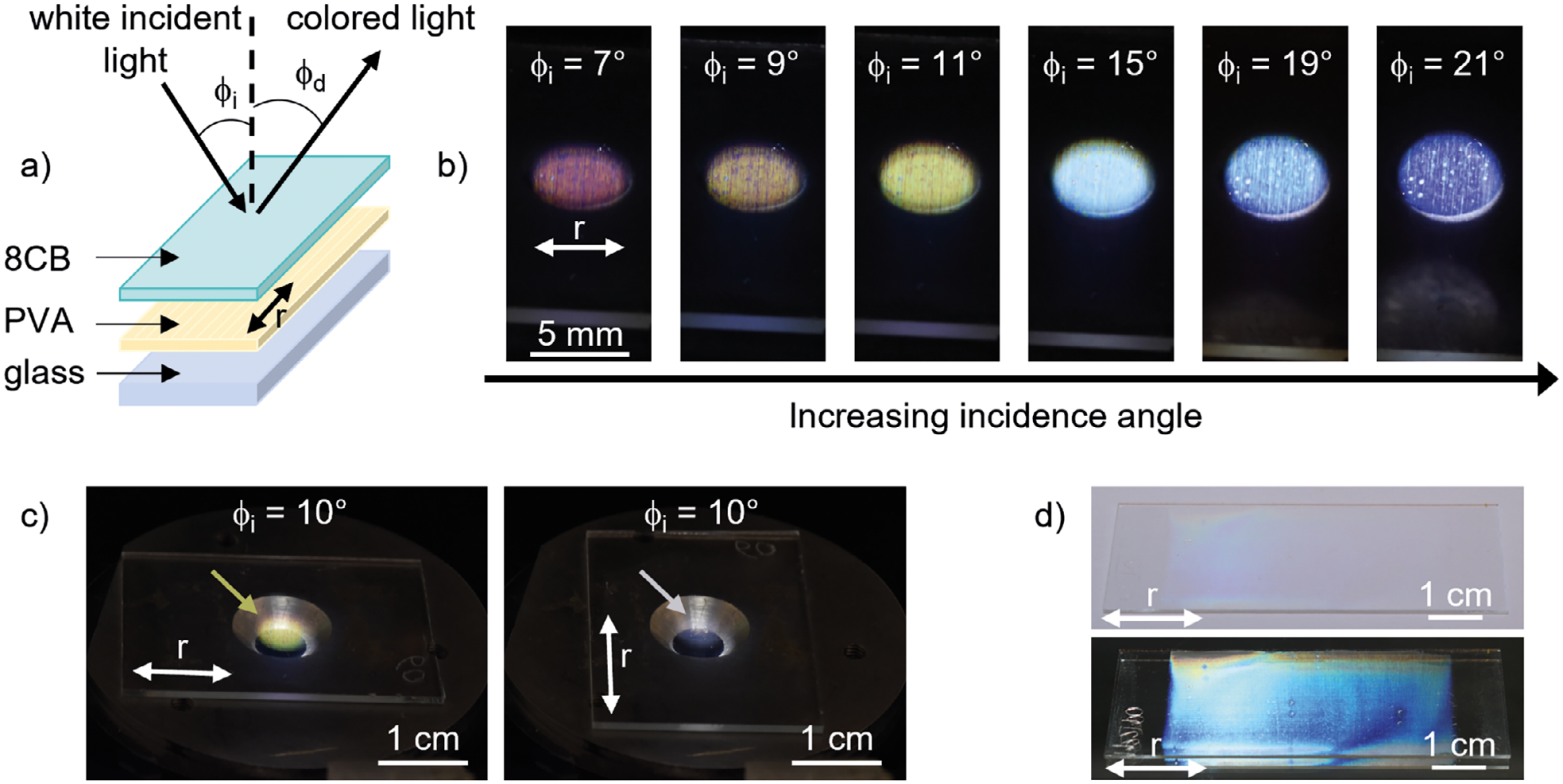
Observation of structural colors from 8CB films observed in reflection. (a) Structure of the sample and geometry used to observe the structural colors. r is the rubbing direction. (b) Structural colors observed as the incidence angle increases (the total angle ϕ_i_ + ϕ_d_ remains constant). Scale bar: 5 mm for all photographs. (c) Effect of the sample orientation. When the plane of incidence is perpendicular to the rubbing direction (left), the sample displays structural colors (yellow here), but when the plane of incidence is parallel to the rubbing direction (right), no colors are observed, whatever the incidence angle. (The single‐headed arrows point to the illuminated part of the film.) (d) Qualitative comparison of the structural colors when the sample is placed either on a white background (top photograph) or on a mirror (bottom photograph), under the same lighting (D65 light booth) and camera recording conditions. The structural colors are clearly enhanced using the mirror background instead of the white background. (In all Figures, the thickness of the liquid crystal films ranges from 0.5 to 1.0 µm.).

Samples showing homogeneous structural colors over several cm^2^ were prepared by using the simple spin‐coating process described in the Section 4. When the sample was examined in reflection (Figure 1a), the whole range of visible colors, from red to blue, was observed as the incidence angle, ϕ_i_, of the incoming light was increased (Figure 1b). Structural colors were also observed in transmission (Figure S1). The iridescent colors were observed only when the plane of incidence was approximately perpendicular to the rubbing axis (Figure 1c and Figure S1). This demonstrates that the structural colors result from light interference occurring perpendicularly to the rubbing axis. Moreover, placing the sample on a mirror strongly enhances the structural colors by improving light reflection (Figure 1d). The samples have not shown any signs of aging, and the structural colors have remained stable for nearly two years.

The samples consist only of a thin, about 1 µm thick, layer of 8CB liquid crystal in its smectic A phase at room temperature, under hybrid anchoring conditions (i.e., the PVA layer provides planar uniform anchoring at the substrate and the 8CB/air interface induces homeotropic anchoring, Figure 1a) [28]. Since the samples do not contain any dye or pigment, the colors they show are structural colors, and since the smectic A period is of only ≈ 3 nm [29], the colors cannot be due to the layered structure of the films. Therefore, they are probably related to their texture, i.e., to the topological defects typical of the smectic A phase. To prove this assumption, we studied the textures of the liquid crystal samples subjected to these hybrid anchoring conditions by polarized‐light optical microscopy (POM) and further by x‐ray scattering and AFM.

### 2.2 Textures of the Liquid Crystal Films

**FIGURE 2:**
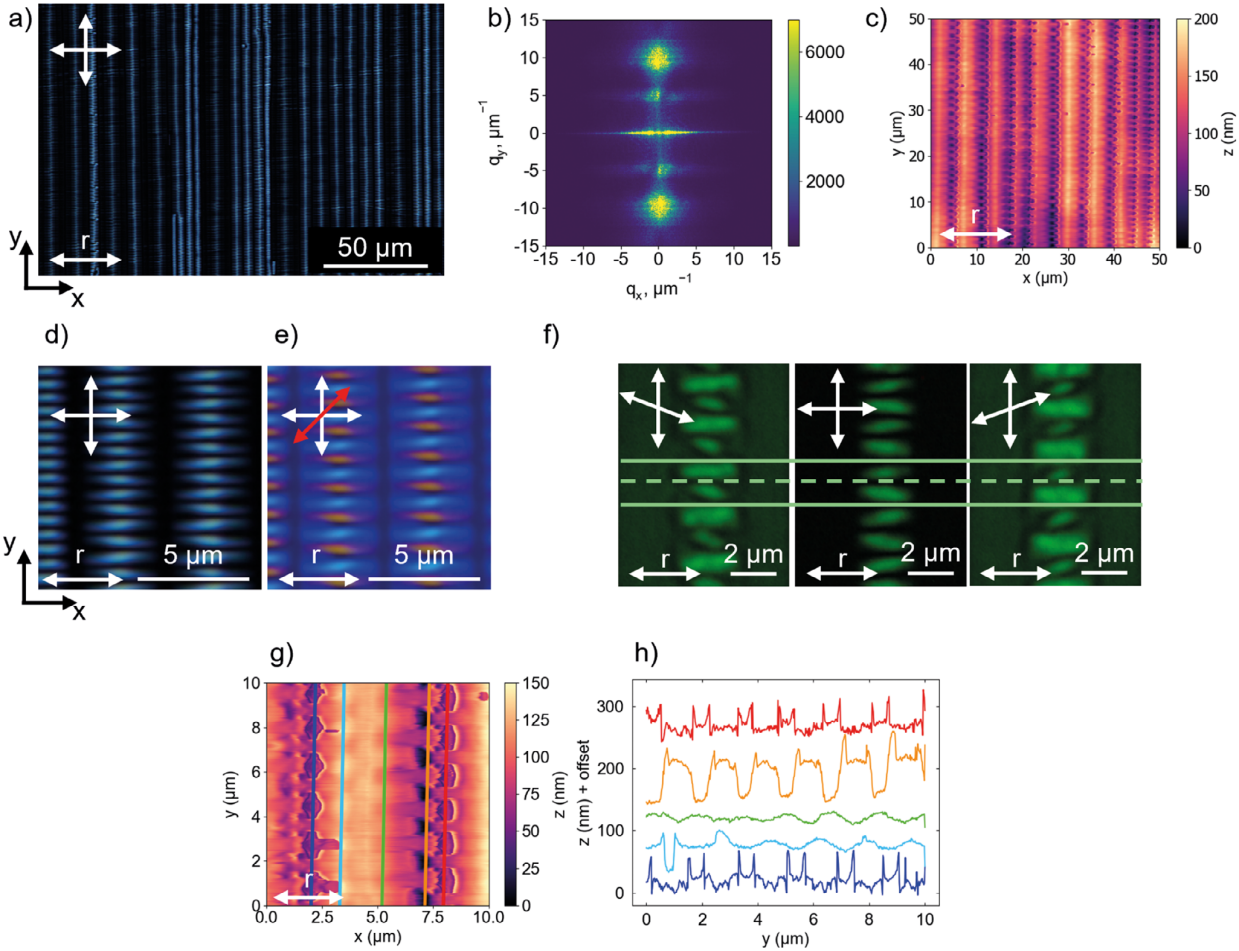
Optical and atomic force microscopy observations of an 8CB film in the smectic A phase at room temperature. (a) Optical texture observed by POM between crossed polarizers (white cross). The rubbing axis is marked r (white double‐headed arrow) and defines the x‐axis. (b) Fourier transform of Figure 2a. (c) Topography of the liquid crystal film observed by AFM. (d) Same sample as in (a) observed at higher magnification. (e) Same sample area as in (d) but observed after insertion of a lambda plate. (f) Contrast changes upon polarizer uncrossing. The green horizontal solid and dashed lines are guides for the eye to assess the intensity of the light transmitted by the “zig” and “zag” domains (see text). Left: analyzer rotated 20° clockwise. Center: crossed polarizers. Right: analyzer rotated 20° counterclockwise. A green interference filter (λ = 546 nm) was used. (g) Enlargement of the area shown in (c). (h) Height profiles, shifted by different amounts for clarity, along the colored straight lines in (g). 8CB film thickness: 0.98 µm.

POM observations indicated that the smectic liquid crystal texture of the samples, over large areas, consists of dark regions, disrupted by many straight linear objects that appear as bright rows, a few µm wide, oriented perpendicular to the rubbing direction (marked x‐axis, Figure 2a). The dark regions between the linear objects are homeotropic areas, as they remain dark when the circular sample stage of the microscope is rotated around the direction of light propagation (which is also the normal to the substrate, marked z‐axis). The linear objects are strongly reminiscent of the linear defects which have been previously studied in great detail in very thin (≈ 100–300 nm) films of 8CB [30, 31], except that they exhibit a periodic striation along their axis (marked y‐axis). Similar striated defects have already been observed in thicker (several micrometers) smectic films [26]. The striation period is fairly well defined along a single linear defect, but it varies slightly from defect to defect. The Fourier transform of the POM image (Figure 2b) of a 1.0 µm thick 8CB sample, shows that the striation period, averaged over the field of view, is 1.2 µm, which is comparable to the wavelengths of visible light. In contrast, no period appears along the x‐axis, which means that the linear defects are not ordered along the rubbing direction. The absence of any periodicity along the x‐axis is consistent with the fact that the structural colors cannot be observed along this direction. The striation of the linear defects observed by optical microscopy was also detected when the topography of the sample was examined by AFM (Figure 2c), which shows that the defect modulation also affects the top surface of the sample. Note that below a critical film thickness (around 0.15 µm), no defect striation could be detected by either POM or AFM (Figure S2).

Different experimental techniques were combined to study the director field in the linear defects in more detail. High‐magnification POM images revealed an alternating pattern of bright and dark lines along the y‐axis (Figure 2d). However, the period along this axis actually appears to encompass two bright lines rather than one, as a faint chevron superstructure was also observed. The presence of the superstructure was qualitatively confirmed by inserting a lambda (wave) plate into the microscope, as the defect bright lines turned into an alternation of blue and orange areas (Figure 2e). The blue (resp. orange) areas correspond to regions where the projection of the director onto the sample plane is approximately parallel (resp. perpendicular) to the slow axis of the lambda plate [32]. Therefore, the projection of the director undulates along the y‐axis, giving rise to a “zig–zag” superstructure. In addition, uncrossing the polarizers (in the absence of the lambda plate) by rotating the analyzer either clockwise or counterclockwise extinguished either the “zig” or the “zag” domains (Figure 2f). This selective extinction can be produced either by opposite twist of the director inside the zig and zag domains (although it should involve both bend and twist of the director, which can only be accommodated in a smectic phase by the occurrence of dislocations [33]) or by an untwisted modulation of the director based on a packing of focal conic domains, as discussed in the Supporting Information text and Figures S3–S6. We also remark that the selective extinction of one or the other domain strongly depends on the focusing of the sample in the microscope—an effect also discussed in the Supporting Information. Whatever the actual structural model, these observations suggest that the periodic striation of the linear defects corresponds to a 3D distortion of the director field and hence of the smectic layers.

AFM images (Figure 2g), recorded at a higher magnification than the POM images, are consistent with the presence of flattened hemicylinders aligned parallel to the y‐axis [23]. Height profiles along straight lines parallel to the y‐axis (Figure 2h) at different positions in the AFM image show that the central part of the hemicylinders is almost flat and probably corresponds to the homeotropic region observed by POM between two adjacent linear defects. In contrast, height profiles recorded between two adjacent hemicylinders show height modulations of about 50 nm, with a period of 1.6 µm that matches fairly well the striation period measured in the POM images.

Finally, the 8CB films were examined by small‐angle x‐ray scattering, in transmission at normal incidence (Figure S7) to study the orientation of the smectic layers thanks to their sharp scattering signal at q_0_ = 1.96 nm^−1^. The smectic period of 8CB (3.17 nm at room temperature) is considerably larger than its molecular length (2.21 nm) [29]. Indeed, due to their strong longitudinal electric dipole moment, 8CB molecules tend to associate in partially overlapping antiparallel dimers which form the smectic layers. Such experiments, in this simple geometry, detect only the domains where the normal to the smectic layers is perpendicular to the x‐ray beam (i.e., parallel to the plane of the substrate). They show that the smectic layers, in the planar regions at the substrate plane, are mostly aligned with their normal along the rubbing axis, with mosaic angular distributions of a few degrees. Furthermore, the alignment improves with decreasing sample thickness (Figure S7). Whatever the sample thickness, no smectic layers are aligned with their normal deviating much from the x‐axis at the substrate plane. This means that the distortion of the director in the linear defects, from planar (along the rubbing direction) on the substrate to homeotropic at the free surface, involves simultaneous zenithal and azimuthal angular deviations, confirming the 3D character already inferred from the detailed POM observations.

### 2.3 Measurements of the Optical Properties of the 8CB Films

**FIGURE 3:**
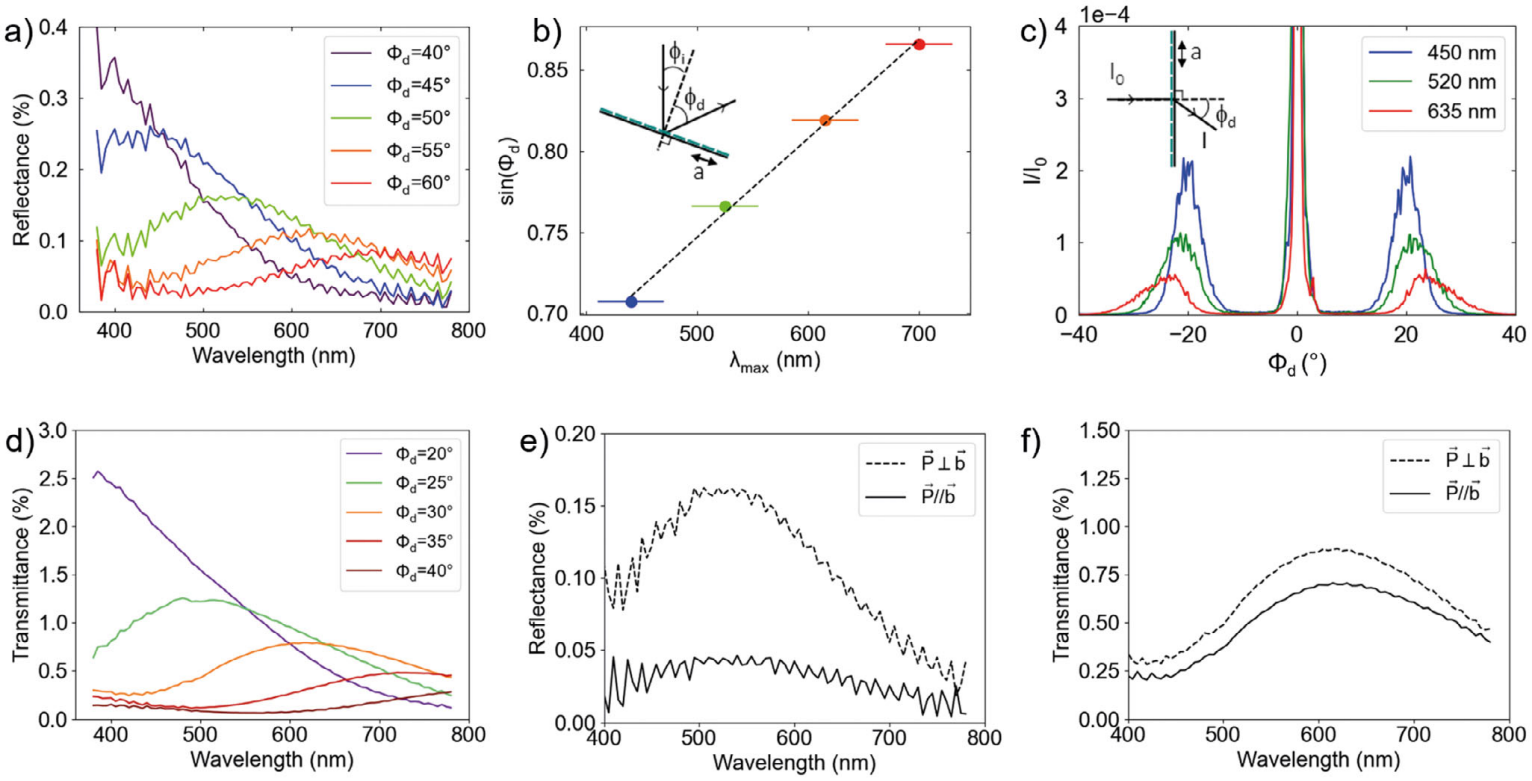
Measurements of the diffraction properties of the 8CB films. (a) Optical spectra in reflection geometry (shown as inset in b), angle of incidence: ϕ_i_ = 20°. (b) sin(ϕ_d_) vs λ_max_. λ_max_ is the wavelength of maximum reflectance extracted from (a). The dashed straight line represents a linear fit of the data (slope: 6.1 × 10^−4^ nm^−1^, intercept: 0.44, the horizontal error bars are due to the large width of the curves in (a)). (c) Laser light diffraction curves at three different wavelengths, in transmission geometry and normal incidence, as shown in the inset. (d) Optical spectra in transmission geometry at normal incidence of light. (e,f) Effect of the polarization of the incident light beam on the optical spectra of an 8CB film in reflection (ϕ_i_ = 20°, ϕ_d_ = 50°) geometry (e) and in transmission (ϕ_i_ = 0°, ϕ_d_ = 30°) geometry (f).

The structural colors of the films, observed qualitatively with the naked eye (Figure 1), were also measured by spectrophotometry in both reflection and transmission geometries (Figure 3a,d). For a fixed angle of incidence ϕ_i_, the wavelength of the maximum of reflectance (or transmission), noted λ_max_, increases with the angle ϕ_d_ of the detector. The relation between sin ϕ_d_ and λ_max_ is linear (Figure 3b) and therefore reminiscent of the simple grating equation: sin ϕ_d_ = sin ϕ_i_ + mλ/a, where m is the order of diffraction, λ is the wavelength of the incident light, and a is the grating period. A linear fit of the data with the grating law gives a = 1.6 µm, which is in good agreement with the defect striation period, 1.3 ± 0.1 µm, measured by POM for this film. The small discrepancy between the two measurements could be explained by small variations in the striation period over large areas of the sample.

Laser diffraction experiments were also performed using different laser wavelengths to study in detail the properties of the 8CB films in transmission (Figure 3c). The diffraction angle increases with the wavelength used, as expected from the grating equation (in transmission), which then gives the grating period, 1.45 ± 0.15 µm, in good agreement with the value measured by optical microscopy (1.4 ± 0.2 µm). Moreover, the intensity of the diffraction peak decreases with increasing wavelength. In addition, the full width at half maximum also increases with the wavelength: 5.8° for λ = 450 nm, 7.9° for λ = 520 nm, and 9.0° for λ = 635 nm. The same trends were observed in the reflection geometry (Figure S8). The 2D visible light diffraction patterns of the 8CB films in both transmission and reflection geometries are shown in Supplementary Information (Figure S9). These diffraction patterns are strikingly anisotropic, which strongly suggests that the structural colors originate from the periodic modulation of the linear defects. Note that, in reflection geometry, the diffraction signal is modulated by weak additional interferences that were observed with two independent set‐ups (Figure 3a and Figures S8,S9). The origin of these weak interferences remains unexplained.

The anisotropy of the diffraction phenomenon that gives rise to the structural colors (Figure 1c) was also investigated more quantitatively by shining linearly polarized light on the samples in both reflection (Figure 3e) and transmission (Figure 3f) geometries. In both cases, the diffraction efficiency is higher for light polarized along the main axis of the linear defects, by a factor of ≈ 4 in reflection geometry and ≈ 1.2 in transmission geometry. The sensitivity of the structural colors to the linear polarization of light is, in fact, expected from symmetry arguments because the texture of the 8CB films has very large in‐plane anisotropy. In this respect, these 8CB films differ from classical systems based on cholesteric liquid crystals, which are sensitive to circularly polarized light.

Typical diffraction efficiencies are around 0.1%, but some samples reached efficiencies of up to 1%–2% (see Figure 3d,f). Moreover, as shown qualitatively in Figure 1d, in reflection geometry, the structural colors can be further enhanced simply by placing the sample on a mirror.

### 2.4 Fine‐Tuning the Structural Colors of the 8CB Films

**FIGURE 4:**
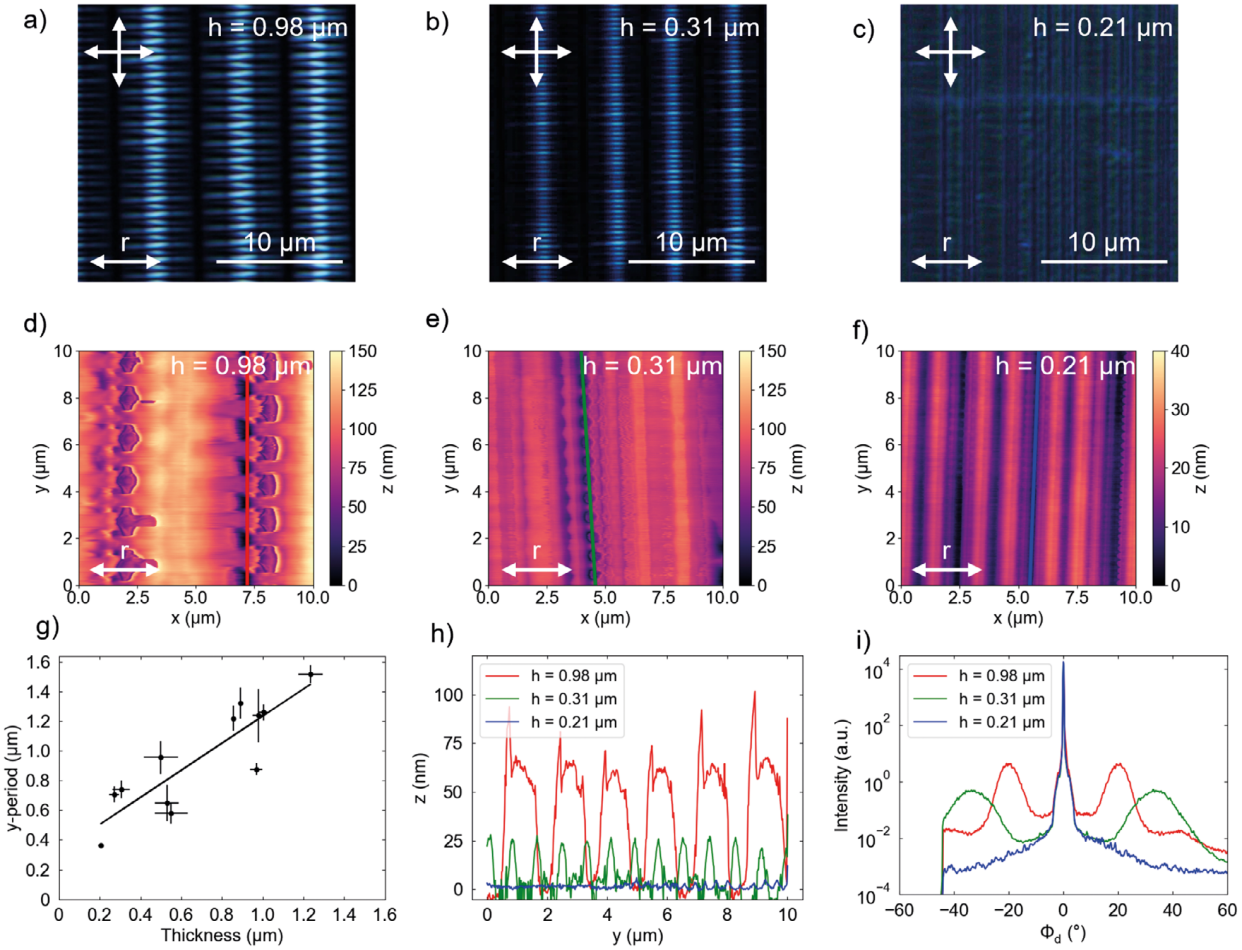
Influence of the thickness, h, on the texture of an 8CB film studied by POM (a–c) and AFM (d–f). The film thickness is shown in the upper right corner. (g) Dependence of the striation period on the film thickness. The solid straight line represents a linear fit of the data (slope: 0.91, intercept: 0.32). (h) Film height profiles along the red, green, and blue lines in (d), (e), and (f), respectively. (i) Laser light diffraction curves (blue laser beam, λ = 450 nm) by the three samples shown in (a–c), in transmission geometry at normal incidence.

The thickness of the 8CB films could be easily adjusted by diluting 8CB with toluene during the spin‐coating step (see Materials and Methods). POM and AFM measurements (Figure 4a–f) showed that the period of the defect striations increases approximately linearly with the film thickness (Figure 4g). Moreover, the height modulation amplitude of the linear defects increases with the thickness (Figure 4h). Laser diffraction experiments in transmission showed that these features affect the optical properties of the film (Figure 4i). As the film thickness decreases, the striation period decreases, and the diffraction peak shifts to wider angles, and eventually disappears. For example, we observed that a 0.98 µm thick film has a striation period of 1.4 µm, resulting in a diffraction angle of 20.2°, whereas a 0.31 µm thick film has a smaller striation period of 0.77 µm, resulting in a larger diffraction angle of 33.7°. These experimental values of diffraction angles are in good agreement with the values 19° and 35.8°, respectively, obtained from the grating equation. The defect striation period of a 0.21 µm thick sample is 0.36 µm, and no diffraction peak is either expected from the grating equation or observed at normal incidence. The intensity of the first diffraction peak increases with the sample thickness. This trend was also observed in the reflection geometry, even for a very thick (8 µm) sample. However, obtaining linear defects with well‐defined striations is much more difficult for thick samples, and they are not well suited for producing structural colors in the visible range because their striation period reaches several microns. Finally, we verified that the strong anisotropy (Figure 1c) of the light diffraction phenomenon giving rise to the structural colors is still observed regardless of film thickness within the investigated range of 0.15 to 1.3 µm.

## 3 Discussion

Classical liquid crystal‐based systems that produce structural colors exploit the large periods arising from the intrinsic order of the phases, i.e., the helical pitch of the cholesteric phase or the smectic period of swollen lamellar phases. The structural colors displayed by the 8CB films described in this report have a completely different origin as they result from the spatial periodic modulation of smectic topological defects, which are not structural but textural features. Previous reports have documented light diffraction by either striped smectic textures induced by temperature gradients or by equidistant uniform linear defects induced by programmable photoalignment [27, 34]. However, in both cases, the distorted texture has a period of several micrometers, which is not convenient for producing structural colors.

The phenomenon of light diffraction giving rise to the structural colors displayed by the 8CB films obeys the simple grating equation, in first approximation, which allows predicting the diffraction angles at which the structural colors appear. This simple, yet well‐defined, behavior suggests that the structural colors are not due to the scattering by disordered heterogeneities (Tyndall scattering) [4] but rather to the constructive interference of visible light scattered by a well‐defined modulation of an effective refractive index along the linear topological defects. The data in Figure 3e,f shows that these interferences depend on the light polarization, which strongly suggests that the phase modulation introduced by the periodic bulk optical axis field plays an important role in the emergence of diffracted modes. However, we emphasize that the surface topography of the smectic layers likely plays an equally important role, because the associated optical phase difference due to the film thickness modulation is of the same order of magnitude ≈ π/4–π/2 for visible wavelengths. The quantitative description of the optical diffraction measurements would therefore require accurate modelling of both the smectic bulk and surface, which goes beyond the simple geometrical model shown in the Supplementary Information and remains as an open challenge. Nevertheless, we emphasize that the simple grating equation used here to relate diffraction angle to wavelength remains valid regardless of the actual values of the diffracted mode amplitudes, since it only expresses a transverse phase‐matching condition between different optical Fourier modes. In a way, the situation is very similar to the classical calculation of reflected waves at a planar interface between two isotropic media or to the more complex observations of reflected waves from liquid crystal solitons [35], for which only the Fresnel coefficients depend on the details (refractive indices, optical axis, etc.) of the localized structure.

Our experimental study strongly suggests that the structural colors are due to light diffraction by the modulated linear defects. As a matter of fact, heating the samples to the nematic or isotropic phase makes the structural colors vanish (Figure S10). The appearance of linear topological defects in the textures of smectic A films under hybrid anchoring conditions has been reported by several groups who determined the organization of the smectic layers for different ranges of film thickness, h. For example, Lacaze et al. studied the linear defects in great detail, mostly in very thin films (h < 500 nm), where they observed a periodic ordering, parallel to the rubbing axis, of uniform defect lines that they described in terms of flattened hemicylinders [23]. In addition to their lack of modulation along the defect axis, we found that these samples are in fact too thin to produce strong structural colors. Topological defects have also been studied in much thicker samples (h > 5 µm) by Ryu et al. and Luo et al. [26, 36]. They observed two kinds of periodicities: one between defects along the rubbing direction and one along the perpendicular direction. However, both are too large to conveniently produce interference of visible light. Moreover, they proposed a description of the smectic layers based on elliptic‐hyperbolic focal conic domains. Similarly, smectic A liquid crystals, confined in microchannels under hybrid anchoring conditions, also exhibit focal conic domains [37, 38]. In fact, the smectic texture can even be switched from an array of focal conic domains to modulated linear defects by applying an in‐plane electric field to 5 µm thick LC cells treated for hybrid anchoring [39]. Similar to the linear defects discussed here, the light diffraction by hexagonal arrays of focal conic domains has already been reported, but due to the large lattice parameter (≈ 5–10 µm), no structural colors were noticed [40]. These arrays can be used to confine gold nanoparticles [41], which could enhance optical contrast and therefore diffraction efficiencies. For the 8CB films with a thickness ≈ 1 µm investigated here, the period of the defect modulation is also close to 1 µm, which makes the determination of the internal structure of the defects by optical microscopy quite challenging. However, as shown in Figure S3, the linear defects observed in our samples seem to have a similar organization of discontinuity surfaces to those observed by Ryu et al. and Luo et al. [26, 36]. This suggests that a description in terms of focal conic domains may also apply to our system despite the much smaller coating thickness. However, as explained in the Supporting Information, the fine details of such packings of focal conic domains are not yet fully understood. Indeed, none of the models used to describe these packings (including ours discussed in theSupporting Information) perfectly respects the homeotropic anchoring at the SmA/air interface, despite its dominant contribution to the total energy.

Our approach to producing structural colors by simply spin‐coating 8CB onto a glass slide covered with a rubbed PVA alignment layer has several advantages over more traditional methods. One major advantage is the use of 8CB, an extremely common and inexpensive liquid crystal that shows, at room temperature, a smectic A phase, one of the simplest liquid crystal phases. Since 8CB is an achiral commercial molecule, this method eliminates the purification steps of natural polymers, such as cellulose or chitin, as well as the asymmetric syntheses of chiral organic compounds. It also dispenses with the high‐temperature syntheses of colloidal mineral nanoparticles and the complex syntheses of block‐copolymers. Moreover, the structural colors can be precisely tuned by simply adjusting the thickness of the 8CB film through dilution of the solution used for spin‐coating. Such tunability can be achieved with molecular cholesteric systems by adjusting composition or temperature, or by applying small voltages. However, most other liquid crystal‐based systems can only be tuned by changing physicochemical parameters, such as nanorod concentration [20], ionic strength [19], water content [42], or chiral dopant concentration [5], which is likely to alter their other physical properties. Another advantage of our process is that it can easily be scaled up to the much larger areas required for applications by replacing spin coating with dip or blade coating. A last original feature of this structural color system is that, unlike cholesteric‐based systems, it is sensitive to linear polarization of light rather than circular polarization. This could be useful, for example, for the head‐up display technology that is being implemented in the automotive vehicle industry, but currently suffers from “ghost” images [43]. To solve this problem, the WaVista system, marketed by Fujifilm, is based on cholesteric liquid crystals, but the use of these 8CB films would eliminate the need for a λ/4 layer.

Compared to more traditional methods of producing structural colors, the approach described here suffers from several drawbacks. While the intensity (up to 1%–2%) of the structural colors is comparable to that of some other colloid‐based systems reported in previous studies [18, 42], it needs to be improved to reach the efficiency (10%–20%) required for practical applications. This could be achieved by using a liquid crystal with higher birefringence or by adding a broadband absorber, a common strategy for this purpose [44]. Figure 1d also demonstrates that the structural colors can be greatly enhanced by depositing the liquid crystal film onto a reflective metallic layer, a straightforward step in glass technology. More vivid structural colors could also be obtained by increasing the thickness of the 8CB film, but the defect striation period would also increase (Figure 4). Then, to keep light diffraction within the visible spectrum, either the anchoring layer or the liquid crystal compound could be modified to adjust the dependence of the striation period on the thickness, as recently demonstrated [23]. The spectral width of the structural colors of the 8CB films, as quantified by the full‐width at half‐maximum (FWHM), has a rather large value of ≈ 200 nm, compared to those (≈ 100‐150 nm) usually reported for more classical systems. Although this criterion is considered less stringent than that of diffraction efficiency [14], it should be improved by using more careful, dust‐free, film deposition conditions in a clean room, which should result in a more uniform film thickness. Finally, unlike chiral self‐assembled systems, our process cannot be easily used to produce free‐standing films that can be handled conveniently. Indeed, due to the use of 8CB, a thermotropic smectic liquid crystal at room temperature, the films have poor mechanical properties and are sensitive to temperature variations. This issue can be addressed by polymerizing the system under UV light, as we have previously shown [45, 46] or by using the liquid crystal film as a template for another substrate [47, 48].

## 4 Materials and Methods

### 4.1 Sample Preparation

The 8CB (4‐octyl‐4‐biphenylcarbonitrile, CAS number: 52709‐84‐9, SmA 32.4°C N 39.5°C I) liquid crystal (by Tokyo Chemical Industry) was used without further purification.

The samples were prepared by a two‐step spin‐coating process. First, standard microscope glass slides were cleaned with a 2 v/v% RBS 25 solution, rinsed with distilled water, and dried under a nitrogen gas flow. Then, a 400 nm thick polymer anchoring layer was deposited on the slides as follows: a 9.1 wt.% aqueous solution of poly(vinyl alcohol) (PVA, Mowiol 4‐98, M_w_ = 27 000 g/mol, CAS: 9002‐89‐5) in distilled water was spin‐coated at 4000 rpm during 60 s. To achieve uniform planar anchoring, the polymer layer was rubbed using a benchtop rubbing machine (Holmarc, HO‐IAD‐BTR‐01) with a roller rotation speed of 500 rpm and a sample stage translation speed of 1 mm/s. Finally, the liquid crystal layer was deposited by spin coating (1000 rpm, 60 s). The thickness of the liquid crystal layer was precisely controlled by diluting 8CB with toluene. Liquid crystal thicknesses ranging from 0.1 to 1 µm were achieved using volume ratios (V_8CB_/V_toluene_) ranging from 0.02 to 0.2. The liquid crystal layer thickness was measured by making a step in the film and measuring the step height using an optical profilometer (ZYGO, Newview 9000). To obtain well‐ordered textures, the samples were heated to the nematic phase and cooled down to room temperature at 0.1°C/min. The samples were then placed in closed Petri dishes and stored under a hood to avoid contamination by dust particles.

(Note that solvents less toxic than toluene have also been tested, and proof of concept was obtained by replacing toluene with methyl isobutyl ketone and n‐butylacetate.)

### 4.2 Optical Characterization

The samples were placed on a small homemade goniometer mounted on the rotating stage of an Olympus BX51 upright microscope. They were illuminated in reflection mode using its LED light source (CoolLED pE‐300lite) through a 5X Olympus objective (LMPLFLN 5X, NA = 0.13). The goniometer was used to tilt the samples to adjust the angle between their normal and the (vertical) direction of the incident light. The colors of the samples were captured directly with a photographic camera equipped with a macro lens.

Optical spectra in reflection and transmission, varying the detector angle, were recorded using a spectrophotometer (Lambda 1050+, Perkin‐Elmer) equipped with a total absolute measurement system (TAMS) accessory. Preliminary experiments performed in situ, under the microscope, using an Ocean Optics spectrometer controlled by home‐made software, to directly probe the spectroscopic properties of selected sample areas, gave data similar to those obtained with the spectrophotometer. These preliminary experiments showed that the samples, several cm^2^ in area, are homogeneous.

Laser diffraction experiments were carried out in both transmission and reflection geometries using a light scattering instrument (OMS4, OPTIS) at three different wavelengths (450, 520, and 635 nm).

### 4.3 Structural Characterization

The optical textures of the samples were observed by polarized light optical microscopy (POM) using the same Olympus BX51 upright microscope (but in transmission) equipped with an Olympus LED light source (CoolLED pE‐300lite), an Olympus U‐POC‐2 (NA: 0.9) condenser, and an X100 objective (Olympus, MPlanFLNBD, NA = 0.9). The textures were captured with an sCMEX20 Euromex camera equipped with a CMOS sensor. The periods of the linear defects were measured by fast Fourier transform of the texture images.

The topography of the samples was characterized by atomic force microscopy (AFM), using an ICON instrument (Bruker) in tapping mode, at room temperature. A Tap300AI‐G AFM probe (Budget Sensors) was used, and the instrument frequency was set at 275 kKz.

Small‐angle x‐ray scattering experiments were performed at the Swing high‐flux beamline of the Soleil synchrotron radiation facility (St‐Aubin, France). q is the scattering vector modulus, q = 4πsinθ/λ, where λ is the wavelength (λ = 0.77 Å) and 2θ is the scattering angle. To minimize the x‐ray absorption by the glass, the samples were prepared using 150 µm thick glass slides (coverslips) instead of standard microscope glass slides.

## Author Contributions

C.N.M. and P.D. designed the project; C.N.M. and R.Z. acquired the data; C.N.M., G.P., A.F., V.S., I.D., C.M., and P.D. analyzed and interpreted the data, C.N.M. prepared the Figures, C.N.M. and P.D. wrote the first article draft, which was edited and reviewed by all the authors.

## Funding

This work was funded by a joint Ph.D. grant from Agence Nationale Recherche et Technologie and Saint‐Gobain Research Paris. The authors thank the Agence Nationale de la Recherche (grant number ANR‐23‐CE24‐0006‐03, project DISPLAY) for financial support. R. Z. and V. S. thank Saint‐Gobain Research Paris and Université de Picardie Jules Verne, respectively, for financial support.

## Conflicts of Interest

Authors declare that they have no competing interests.

## Supporting information




**Supporting File**: advs76646‐sup‐0001‐SuppMat.pdf.

## Data Availability

All data are available in the main text or the supplementary materials.
