# Supplementary material for: Structural Colors from Modulated Topological Defects in Molecular Smectic Liquid Crystals
Source: Adv Sci (Weinh). 2026 Jul 30:e76646. Online ahead of print. doi: 10.1002/advs.76646 (PMC13423482; doi:10.1002/advs.76646)
Supplement: Supplementary file 1 — Supporting File: advs76646‐sup‐0001‐SuppMat.pdf. [file ADVS-9999-e76646-s001.pdf]

Supporting Information for

Structural colors from modulated topological defects in molecular smectic liquid crystals

C. N. Mahyaoui, G. Poy, R. Zehra, A. Fouques, V. Sergan, I. Dozov, C. Meyer and P. Davidson

Authors for correspondence:
Camille N. Mahyaoui: camille.mahyaoui@saint-gobain.com
Guilhem Poy: guilhem.poy@umontpellier.fr
Patrick Davidson: patrick.davidson@universite-paris-saclay.fr

This PDF file includes:

Supporting text
Figs. S1 to S10
SI References

Supporting Information Text

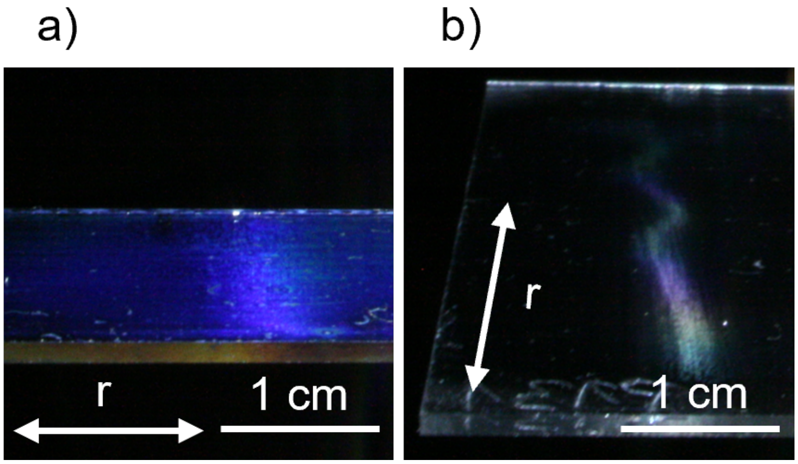

Fig. S1. Observation and properties of structural colors in transmission geometry; influence of the angle between the plane of incidence and the rubbing direction. (a) plane of incidence perpendicular to the rubbing direction; (b) plane of incidence parallel to the rubbing direction (the faint curved streak on the right is an illumination artefact).

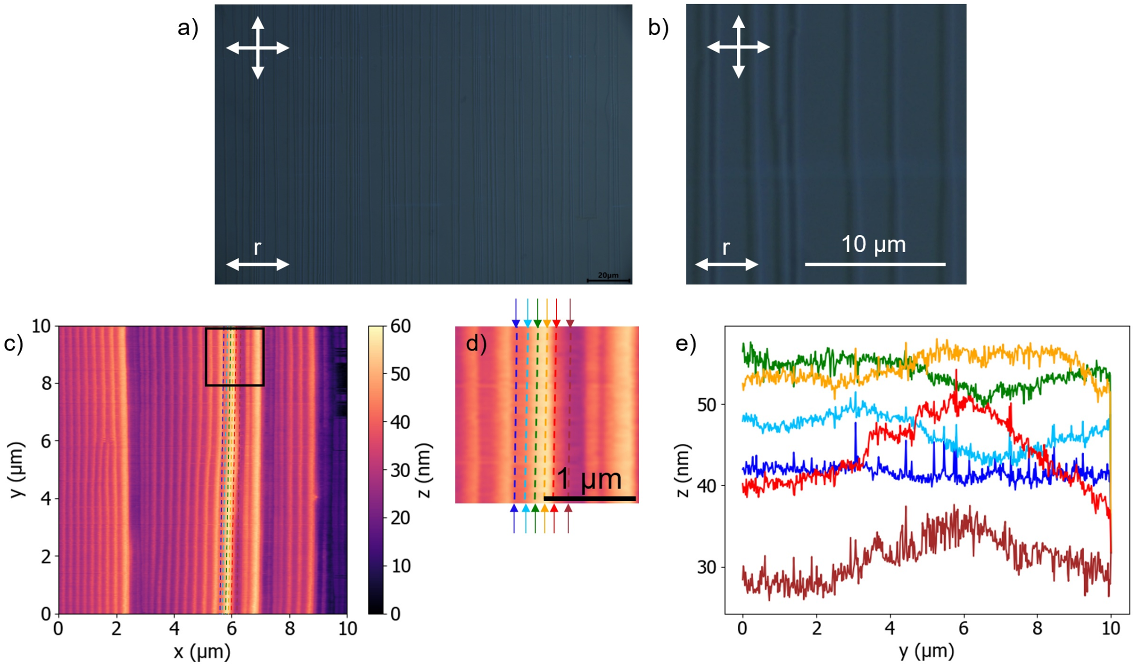

Fig. S2. Polarized light microscopy (POM) and atomic force microscopy (AFM) study of a 120 nm thick 8CB coating. (a,b) POM images at two different magnifications. The white cross represents the polarizer and analyzer axes, and the double-headed arrow, r, is the rubbing direction; (c) AFM image of the same coating; (d) Magnified view of the square box in (c); (e) Topographic profiles of the coating along the vertical straight lines shown in (c). No linear defect striations are visible on these images. Note that the vertical scale is 10 times smaller compared to Fig. 2h of the main text, making the AFM noise much more conspicuous.

1. Optical modeling of microscopy images

We tried to model the microscopy images of the modulated linear defect described in the main text, based on two tentative models for the optical axis field described in the next two subsections and optical microscopy simulations described in the final subsection.

A. Model based on a modulated twisted optical axis field. The experimental optical micrographs (POM) in the main text show that, observed between crossed polarizers, the texture is composed of bright zigzag bands. Furthermore, by uncrossing the

analyzer, we observed that one of the two band families (either the zig or zag bands) can be almost extinguished—although we remark again that this extinction depends on the focusing of the sample in the microscope. One of the possibilities to explain these observations is that the zig and zag domains are associated with twisted optical axis fields of opposite handedness, as described by the following optical axis field along the yz-plane centered in the middle of the modulated smectic texture:

$$\boldsymbol{n} = \begin{pmatrix} \sin\alpha\cos\beta \\ \sin\alpha\sin\beta \\ \cos\alpha \end{pmatrix} \quad [1]$$

with $\alpha = \pi/2(1 - z/L_z)$ the director polar angle and $\beta = \tau_0 \cos[2\pi y/L_y]\, z/L_z$ the azimuthal angle. Here, L_z is the layer thickness (assumed constant for simplicity) and L_y is the modulation period along the y-axis. This director field is nonplanar and smoothly interpolates between the planar anchoring at the substrate $z = 0$ and the homeotropic SmA/air interface at $z = L_z$. We remark that twist in a SmA can only be explained by the presence of screw dislocations. Instead of fully specifying the layer structure of the smectic and the density of these screw dislocations (which is very challenging), we focus here on the very simple Eq. 1 to evaluate the optical properties of the layer.

To gain insight into the optical properties of this director field, one can try to apply the Ong formula (1) for the light transmission in a twisted director structure with total twist angle $\tau = \tau_0 \cos[2\pi y/L_y]$ and Mauguin number $M = \pi L_z \Delta n_{\text{eff}}/(\tau\lambda)$ (with λ the wavelength of light and Δn_{eff} an effective birefringence for the layer):

$$T = \frac{\sin^2\Phi}{1+M^2} + \sin(\tau+\phi_a)\left\{\left[1 - \frac{2\sin^2\Phi}{1+M^2}\right]\sin(\tau+\phi_a) - \frac{\sin(2\Phi)\cos(\tau+\phi_a)}{\sqrt{1+M^2}}\right\}, \quad [2]$$

with $\Phi = \sqrt{\tau^2 + (\eta/2)^2}$ an effective retardance and $\eta \equiv 2M\tau = 2\pi\Delta n_{\text{eff}} L_z/\lambda$. Similar to our experiments, we assumed in this formula that the polarizer (resp., analyzer) is at 90° (resp., at an angle ϕ_a) with respect to the rubbing direction, and also that the director is perfectly anchored along the rubbing direction on the substrate. Assuming $M^2 \gg 1$, we deduce from this formula that the extinction of a twisted domain at positions $y = kL_y$ (with k an arbitrary integer) should happen when the analyzer angle compensates the maximal twist angle, i.e. when $\phi_a = -\tau_0$, or equivalently at positions $y = (k + 1/2)L_y$ when $\phi_a = \tau_0$. In the next sections, we will evaluate with our optical modeling software *Nemaktis* whether this observation holds true despite the non-planarity of this director field model—quasi-planarity being one of the assumptions for the validity of the Ong formula.

B. Model based on an untwisted optical axis field modulation. Alternatively, one may also try to explain our experimental observations with a model based on an untwisted optical axis field. Indeed, let us first consider with Jones calculus the optical transmittance T between polarizer at 90° and analyzer at angle ϕ_a of an effective birefringent layer of thickness L_z and in-plane effective optical axis $\boldsymbol{u} = \cos\beta\,\boldsymbol{e}_x + \sin\beta\,\boldsymbol{e}_y$ (with $\beta(y) = \beta_0\cos(2\pi y/L_y)$ and L_y the modulation period along y):

$$T = \sin^2(\phi_a)\cos^2\frac{\eta}{2} + \sin^2(2\beta - \phi_a)\sin^2\frac{\eta}{2} \quad [3]$$

with η the birefringent layer retardance. With $\phi_a = 0$, one therefore obtains bright bands at positions $y = kL_y/2$ (with k an integer), and with $\phi_a = 2\beta_0$ (resp., $\phi_a = -2\beta_0$), the bands at positions $y = kL_y$ (resp., $y = (k + 1/2)L_y$) are extinguished.

Motivated by this theoretical observation, we tried to construct a fully three-dimensional model of the layer structure and optical axis field compatible with such a modulation. Fig. S3a shows an experimental dark-field reflection micrograph of the linear structure discussed in the main text. This image suggests the existence of periodic surfaces of discontinuity separating SmA domains (homeotropic layers outside the linear structure, tilted layers inside), as schematically shown in Fig. S3b. In fact, this arrangement of domains is reminiscent of a work by Luo *et al.* (2) describing similar linearly-modulated texture in thicker smectic slabs. Similar to their model, we assume that each elementary quasi-polygonal domain in Fig. S3b is associated with a focal conics domain (FCD), but with a different orientation of the generating ellipse and hyperbola, as shown in Fig. S3c.

The optical axis field inside each quasi-polygonal domain is constructed from the generating ellipse and hyperbola as follows. For simplicity, we assume that the spatial origin coincides with the center of the ellipse. Each point of the ellipse (resp. hyperbola) is parametrized as $\boldsymbol{p}_e = \cos(u)\boldsymbol{e}_a + \sin(u)\boldsymbol{e}_b$ (resp., $\boldsymbol{p}_h = \boldsymbol{h}_c/\cos(v) - \tan(v)\boldsymbol{h}_b$). The vectors $\boldsymbol{e}_{a,b}$ and $\boldsymbol{h}_{c,b}$ defining the ellipse and hyperbola are shown on Fig. S3c, and have the following properties: $|\boldsymbol{e}_a| = a$, $|\boldsymbol{e}_b| = |\boldsymbol{h}_b| = b$, $|\boldsymbol{h}_c| = c \equiv \sqrt{a^2 - b^2}$, $\boldsymbol{h}_c \parallel \boldsymbol{e}_a \perp \boldsymbol{e}_b$, and $\boldsymbol{h}_b \perp \boldsymbol{e}_{a,b}$, with a (resp., b) the long (resp., small) axis of the ellipse, and c its linear eccentricity.

Each point along the line crossing $\boldsymbol{p}_e$ and $\boldsymbol{p}_h$ can be written as $\boldsymbol{p} = d\,\boldsymbol{n} + \boldsymbol{p}_m$, with:

$$\boldsymbol{p}_m \equiv \frac{a\,\boldsymbol{p}_h - c\cos(u)\cos(v)\boldsymbol{p}_e}{a - c\cos(u)\cos(v)}, \quad [4]$$

$$\boldsymbol{n} \equiv \frac{\cos(v)\,[\boldsymbol{p}_h - \boldsymbol{p}_e]}{a - c\cos(u)\cos(v)} = \frac{1}{a - c\cos u\cos v}\begin{pmatrix} c - a\cos u\cos v \\ -b\sin u\cos v \\ -b\sin v \end{pmatrix}, \quad [5]$$

where the coordinates of $\boldsymbol{n}$ are given in the orthonormal basis $\{\boldsymbol{e}_a/a, \boldsymbol{e}_b/b, \boldsymbol{h}_b/b\}$. $\boldsymbol{n}$ can be interpreted as the unit* director field for all points on the line crossing $\boldsymbol{p}_e$ and $\boldsymbol{p}_h$, while d parametrizes the same line and can be used to uniquely identify individual smectic layer since varying u and v at fixed d generates a Dupin cyclide.

*A direct calculation of the norm of $\boldsymbol{n}$ shows that it is equal to 1 since $c^2 = a^2 - b^2$.

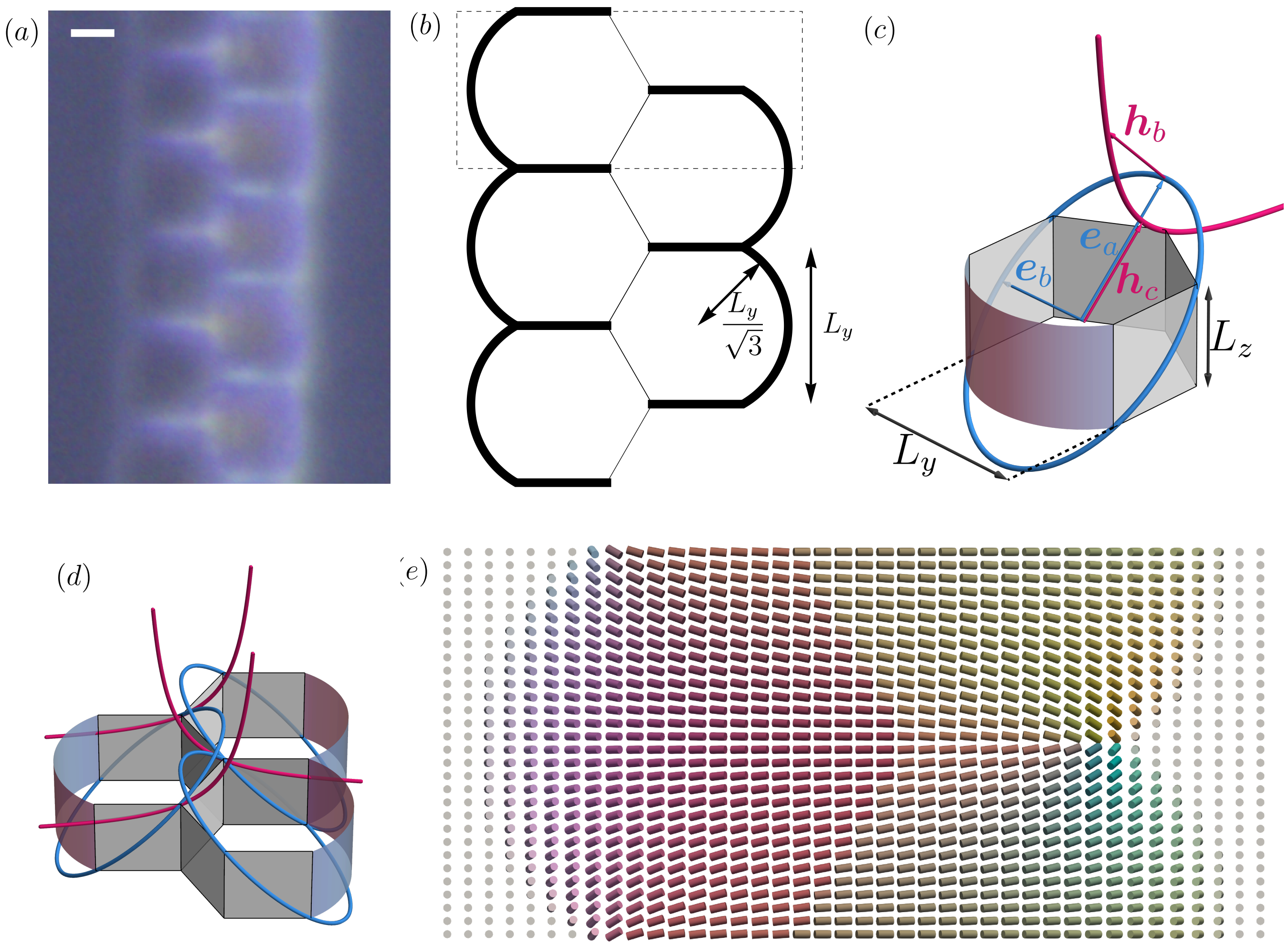

Fig. S3. (a) Dark-field reflection micrograph of the modulated SmA texture. The scale bar represents $1\,\mu\mathrm{m}$. (b) Quasi-polygonal domains trying to mimic the positions of discontinuity surfaces (thick black lines) visible in (a). (c) Ellipse and hyperbola generating a focal conic domain inside each quasi-polygonal domain of (b). (d) Same as (c) for several elementary domains packed as in (b). (e) xy slice of the director field at $z = L_z/4$ inside the dashed domain of (b).

To express the director $\boldsymbol{n}$ as a function of the spatial position $\boldsymbol{p}$, it is enough to calculate the parameters u, v and d as a function of $\boldsymbol{p}$. After some algebra, one finds:

$$\begin{pmatrix}\cos u(\boldsymbol{p})\\ \sin u(\boldsymbol{p})\end{pmatrix} = \frac{2}{|\boldsymbol{p}|^2 + b^2 - d(\boldsymbol{p})^2}\begin{pmatrix}\boldsymbol{p}\cdot\boldsymbol{e}_a - c\,d(\boldsymbol{p})\\ \boldsymbol{p}\cdot\boldsymbol{e}_b\end{pmatrix} \quad [6]$$

$$\begin{pmatrix}\cos v(\boldsymbol{p})\\ \sin v(\boldsymbol{p})\end{pmatrix} = \frac{1}{\boldsymbol{p}\cdot\boldsymbol{h}_c - a\,d(\boldsymbol{p})}\begin{pmatrix}\frac{|\boldsymbol{p}|^2 - b^2 - d(\boldsymbol{p})^2}{2}\\ \boldsymbol{p}\cdot\boldsymbol{h}_b\end{pmatrix} \quad [7]$$

As for the value of $d(\boldsymbol{p})$, it can be found analytically by finding the zeros of the following quartic equation—obtained from the norm of either Eq. 6 or Eq. 7—with Ferrari's method:

$$Q(\boldsymbol{p}, a, b, c, d) \equiv \left[\frac{|\boldsymbol{p}|^2 + b^2 - d^2}{2}\right]^2 - \left[a(\boldsymbol{p}\cdot\boldsymbol{e}_x) - c\,d\right]^2 - \left[b\,(\boldsymbol{p}\cdot\boldsymbol{e}_y)\right]^2 \quad [8]$$

This quartic function admits four zeros, but only one of those is associated with an FCD of type I. The right solution can be selected as follows:

- If $Q(\boldsymbol{p}, a, b, c, c) \leq 0$ (horn cyclide case), $\boldsymbol{d}(\boldsymbol{p})$ = maximal solution below c;
- If $Q(\boldsymbol{p}, a, b, c, c) > 0$ and $Q(\boldsymbol{p}, a, b, c, a) \leq 0$ (ring cyclide case): $\boldsymbol{d}(\boldsymbol{p})$ = unique solution between c and a;
- If $Q(\boldsymbol{p}, a, b, c, a) > 0$ (spindle cyclide): $\boldsymbol{d}(\boldsymbol{p})$ = minimal solution above a.

To simulate the complete director field, we set up a Cartesian grid inside the dashed domain of Fig. S3b, and repeat the above calculation of the director for each point inside a given quasi-polygonal domain (points outside being attributed a director normal to the substrate). We remark that this director field is far from being perfect since the homeotropic anchoring at the SmA/air interface is not perfectly homeotropic in this model, but it nevertheless provides a simple basis for probing the optical properties of this structure. An example of an xy slice of the director field at $z = L_z/4$ is shown in Fig. S3d.

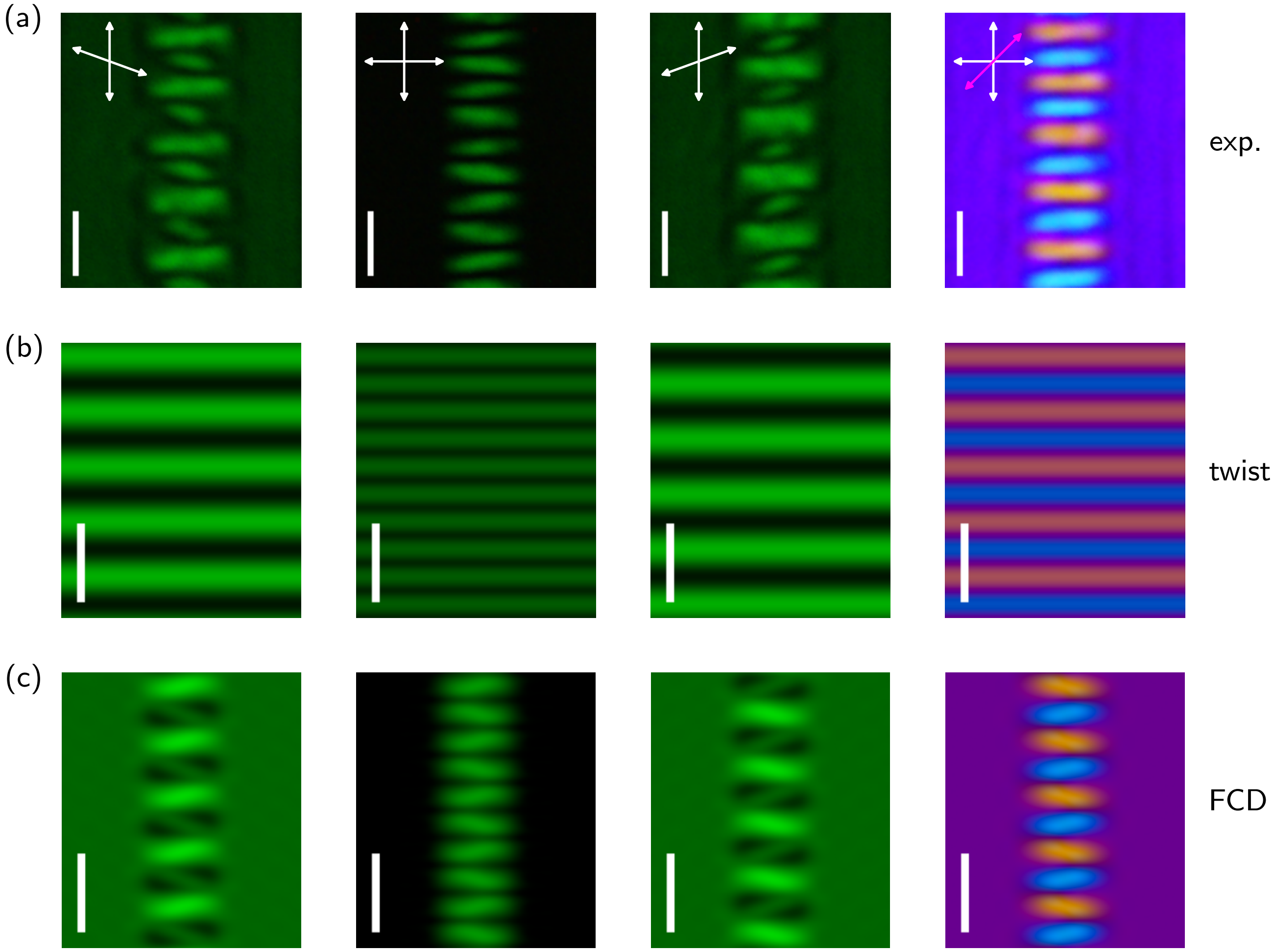

Fig. S4. (a) Experimental polarized light optical micrographs of the modulated linear smectic texture. The polarizer and analyzer (resp., λ-waveplate) orientations are indicated by white (resp., red) arrows. The polarizer is always at 90° with respect to the horizontal axis. (b,c) Simulated micrographs in the same POM configurations as (a) for the twist-modulation model of Sec. I.A. and (b) for the FCD model of Sec. I.B (c). All micrographs were obtained in ideal focusing conditions in the microscope, with the numerical aperture of the condenser set to approximately 0.2. Simulations for the first three columns (resp., last column) were done with a single wavelength at $546\,\mathrm{nm}$ (resp., multiple wavelength in the visible range reproducing the spectrum of a D65 standard illuminant). The white bars represent $2\,\mu\mathrm{m}$.

C. Optical simulation results. We used the open-source microscopy software *Nemaktis* developed by one of us (3) to simulate the optical micrographs associated with both models of the previous subsections. These simulations rely on an efficient beam-propagation-method-based calculation of the optical fields transmitted through the smectic layer, starting from a set of incident plane-waves with wavevectors included in an illumination cone mimicking a Köhler illumination setup. In order to simulate color images, multiple incident wavelengths are also used in order to simulate a typical white light spectrum—assumed here to be the one of a D65 standard illuminant. Once this calculation is done, all transmitted fields are refocused on an imaging plane through an ideal imaging lens that allows us to vary the focusing conditions, and then recombined by intensity since fields from different incident wavelengths or wavevectors are incoherent to each other. This last step can also take into account the presence of any number of polarized microscopy elements.

Fig. S4 shows our results in near-focusing conditions, both for experimental and simulated images. We remark that ideal focusing was determined visually by minimizing the Becke lines near the boundary of the pattern and differed by $1\,\mu\mathrm{m}$ when switching from a single illumination wavelength to white light illumination. As visible, both models correctly reproduce the change of periodicity in the pattern when uncrossing the analyzer with respect to the polarizer, and also correctly reproduce the appearance of blue and orange bands when a λ-waveplate is introduced in the microscope. In addition, the model based on

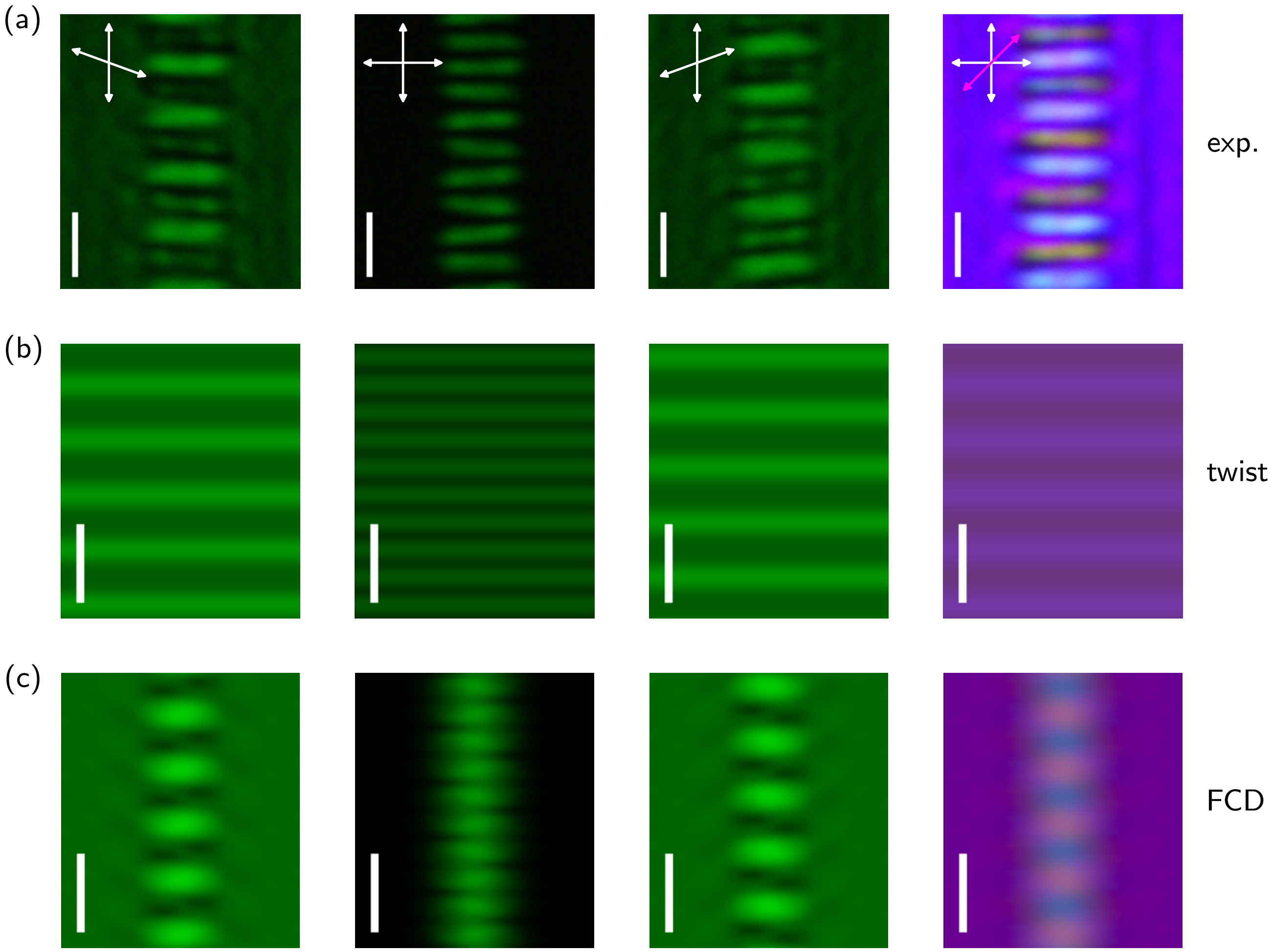

Fig. S5. Same as Fig. S4, but with micrographs captured and simulated $2\,\mu m$ above the focusing plane.

packed FCDs correctly predicts the zig-zag tilt of the bands—something that the twist model cannot predict since it is based on a simple 1D modulation.

Going out-of-focus $2\,\mu m$ above the focusing plane (Fig. S5), we noticed that the contrast of the zig and zag bands in the green experimental POM images was inverted with respect to Fig. S4 when the analyzer is uncrossed. This inversion of the zig-zag band contrast is well reproduced again in both models, although we note that our simulations predict a blurrier pattern than in the experiment for a condenser numerical aperture of 0.2. However, the color images with λ-waveplate are now in disagreement: both models predict an inversion of blue and orange bands $2\,\mu m$ above the ideal focusing plane, but the experimental image shows that the colors should not change with respect to Fig. S4. In fact, the inversion of blue/orange bands does happen in the experiments, but only at a higher defocusing of $4\,\mu m$.

Finally, Fig. S6 shows a comparison of experimental and simulated natural light micrographs, without any polarized microscopy elements and with white light illumination. The twist model incorrectly predicts a vanishingly small intensity modulation—due to the deviation of extraordinary rays by the smectic layers—whereas the FCD-based model fares better, with a correct reproduction of the zig-zag pattern of the experimental image.

Overall, we conclude that the FCD model yields a slightly better agreement than the twist model when comparing with experimental images. However, we emphasize that the FCD model is far from being perfect because it does not yield a perfect homeotropic alignment at the SmA/air interface—although this interface is known to be associated with a very high anchoring energy (4, 5). In principle, both a topographical modulation of this interface and the addition of bulk dislocation lines should allow the system to reach a lower energy state with better anchoring of the optical axis. Such an improved model is out of the scope of this paper but may be an interesting avenue for future research on equilibrium textures in thin SmA coatings.

References

1. HL Ong, Origin and characteristics of the optical properties of general twisted nematic liquid-crystal displays. *J. Appl. Phys.* **64**, 614–628 (1988).

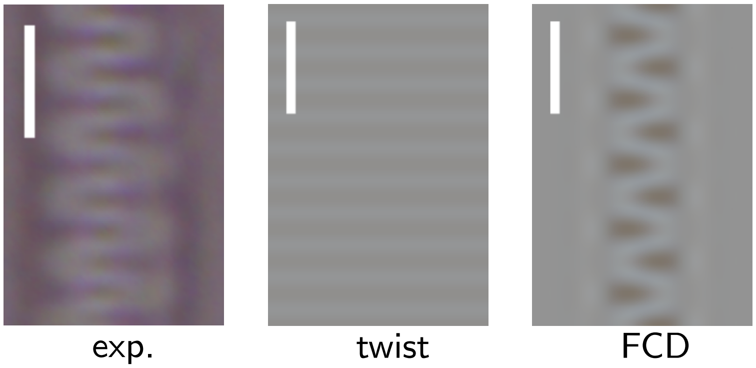

Fig. S6. Natural light micrographs obtained from the experiments (left), the twist model (middle) or the FCD model (right). The white bars represent $2\,\mu m$.

2. D Luo, J Wu, Z Guo, J Xia, W Hu, Generation and regularization of zigzag focal conic domains guided by thermodynamic-driven topological defect evolution. *Giant* **20**, 100327 (2024).
3. G Poy, S Žumer, Physics-based multistep beam propagation in inhomogeneous birefringent media. *Opt. Express* **28**, 24327 (2020).
4. E Lacaze, JP Michel, M Alba, M Goldmann, Planar anchoring and surface melting in the smectic-*a* phase. *Phys. Rev. E* **76**, 041702 (2007).
5. DK Yoon, et al., Liquid-crystal periodic zigzags from geometrical and surface-anchoring-induced confinement: Origin and internal structure from mesoscopic scale to molecular level. *Phys. Rev. E* **82**, 041705 (2010).

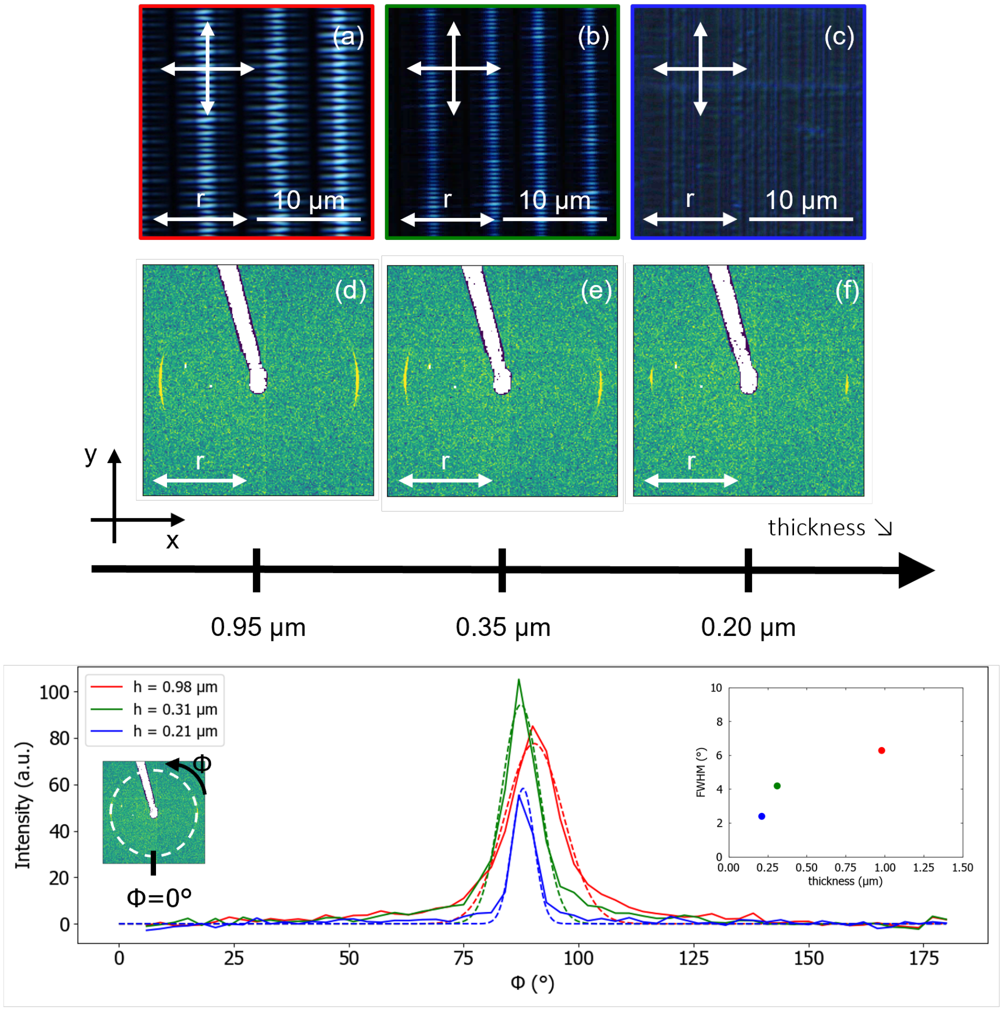

Fig. S7. Dependence on the thickness of the 8CB coating of its texture and its structure. (a-c) Dependence on the thickness of the texture, observed by polarized light microscopy. For a thickness of $h = 200\,\mathrm{nm}$, the linear defect striations are no longer visible. (d-f) Dependence on the thickness of the small-angle x-ray scattering pattern. The mosaicity of the smectic reflection decreases with decreasing thickness, which is due to an improvement of the smectic alignment. Bottom: solid lines: plots of the scattered intensity versus azimuthal angle Φ (see left inset) for the three different thicknesses (top left inset); dashed lines: fits of the data by Gaussians; right inset: plot of the mosaic distribution width (FWHM) versus coating thickness.

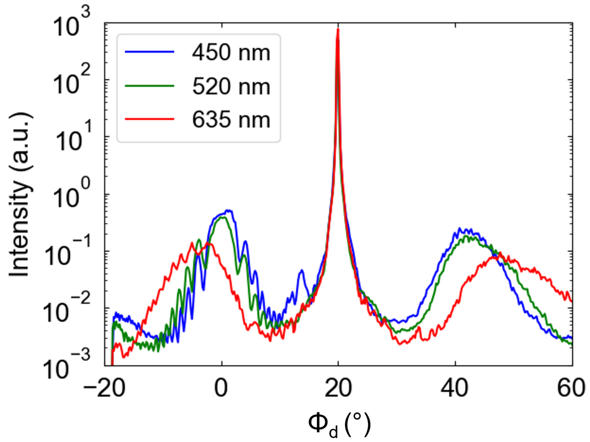

Fig. S8. Laser diffraction patterns of an 8CB coating (thickness $0.98\,\mu\mathrm{m}$) at three different wavelengths, in reflection geometry and at light incidence angle of 20°.

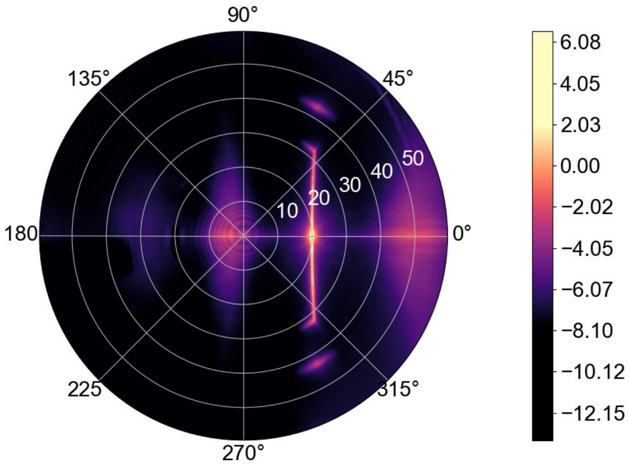

Fig. S9. Two-dimensional visible light diffraction patterns of an 8CB coating in both reflection (left, incidence angle: 20°) and transmission (right, normal incidence) geometries (thickness: $0.98\,\mu\mathrm{m}$, incidence angle 20°).

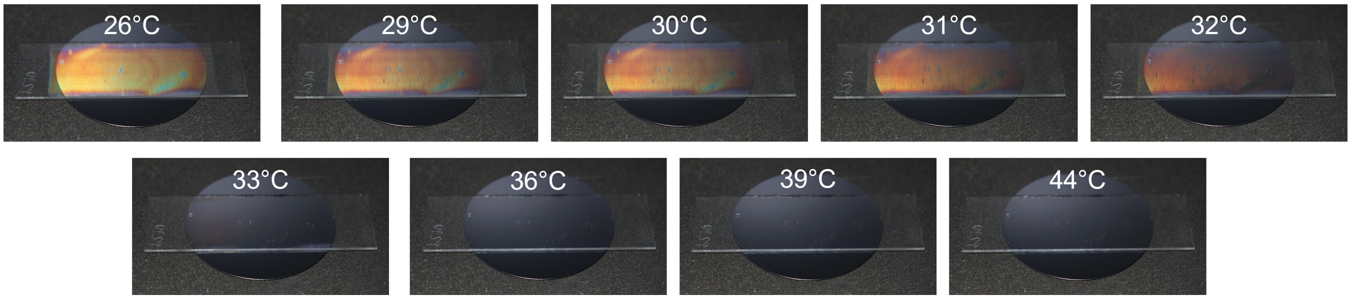

Fig. S10. Series of photographs of an 8CB film, on a glass substrate placed on a mirror (silicon wafer), heated with a heating stage from room temperature (smectic A phase) to 33 °C (nematic phase), and then to 44 °C (isotropic phase). The structural colors first fade slightly and then vanish abruptly at the SmA/N transition.
